# Supplementary material for: Genetic Variation Study of Several Romanian Pepper (Capsicum annuum L.) Varieties Revealed by Molecular Markers and Whole Genome Resequencing
Source: Int J Mol Sci. 2024 Nov 5;25(22):11897. doi: 10.3390/ijms252211897 (PMC11593692; doi:10.3390/ijms252211897)

**Supplementary figure 12.** A visual representation of seven Romanian pepper (*C. annuum* L.) varieties with valuable traits, homologated populations by the Buzău Vegetable Research and Development Station (VRDS), Decebal (gDEC), Vladimir (gVLA), Galben superior (gGAL), Splendens (gSPL), Cosmin (gCOS), Roial (gROI) and Cantemir (gCAN).


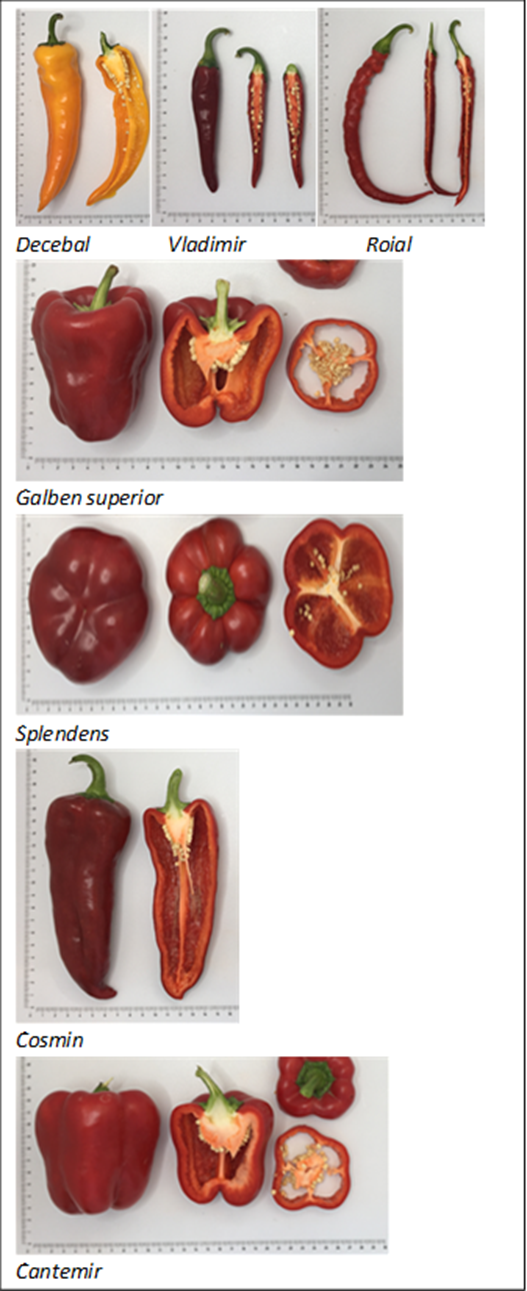

Supplement: Supplementary file 1 [file ijms-25-11897-s001.zip › Supplementary File S12.docx]
