# Supplementary figures and images for: Genetic Variation Study of Several Romanian Pepper (Capsicum annuum L.) Varieties Revealed by Molecular Markers and Whole Genome Resequencing
Source: Int J Mol Sci. 2024 Nov 5;25(22):11897. doi: 10.3390/ijms252211897 (PMC11593692; doi:10.3390/ijms252211897)

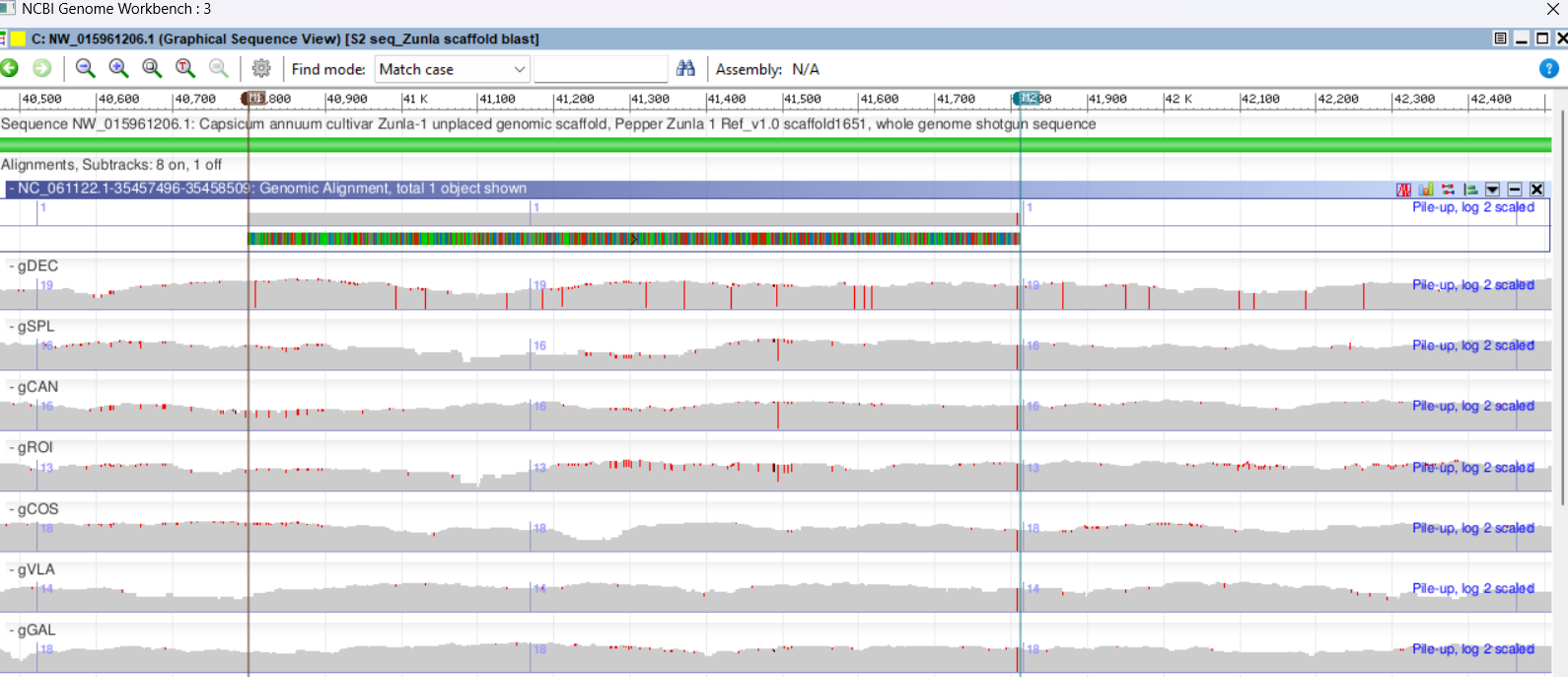

Supplement: Supplementary file 1 [file ijms-25-11897-s001.zip › Supplementary File S10.png]

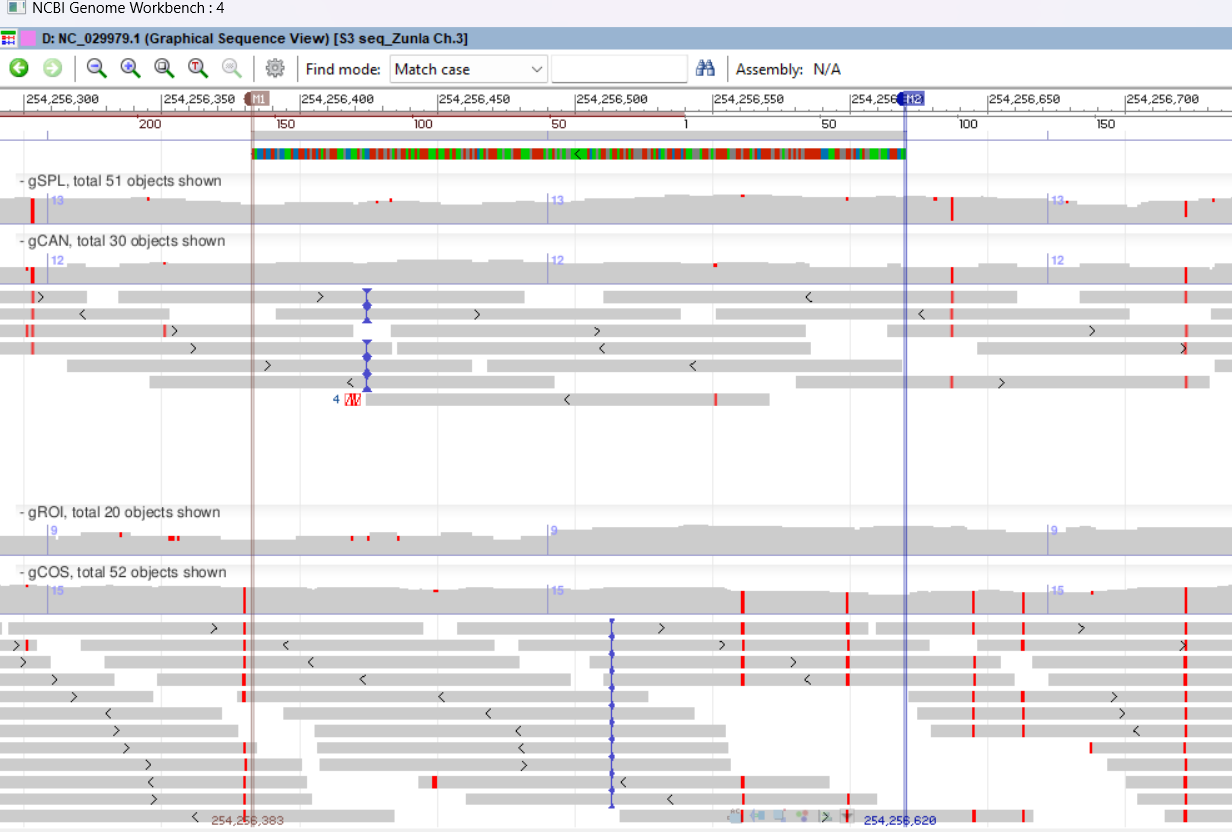

Supplement: Supplementary file 1 [file ijms-25-11897-s001.zip › Supplementary File S11.png]
